# The Addition of α-cyclodextrin and γ-cyclodextrin Affect Quality of Dough and Prebaked Bread During Frozen Storage

**DOI:** 10.3390/foods8050174

**Published:** 2019-05-22

**Authors:** Jianjun Zhou, Yuan Ke, Francisco J. Barba, Shensheng Xiao, Xianqin Hu, Xinguang Qin, Wenping Ding, Qingyun Lyu, Xuedong Wang, Gang Liu

**Affiliations:** 1 Key Laboratory for Deep Processing of Major Grain and Oil (Wuhan Polytechnic University), Ministry of Education, Wuhan 430023, China; 13871500262@163.com (J.Z.); keyuan0720@163.com (Y.K.); xiaoshensheng666@163.com (S.X.); qxg198304@163.com (X.Q.); whdingwp@163.com (W.D.); lqingy2001@163.com (Q.L.); 2 School of Food Science and Engineering, Wuhan Polytechnic University, Wuhan 430023, China; 3 Hubei Key Laboratory for Processing and Transformation of Agricultural Products (Wuhan Polytechnic University), Wuhan 430023, China; 4 Nutrition and Food Science Area, Preventive Medicine and Public Health, Food Science, Toxicology and Forensic Medicine Department, Faculty of Pharmacy, Universitat de València, Avda.Vicent Andrés Estellés, s/n 46100 Burjassot, València, Spain; Francisco.Barba@uv.es; 5 Engineering Research Center of Feed Protein Resources on Agricultural By-product, Ministry of Education, Hubei Key Laboratory of Animal Nutrition and Feed Science, Freshwater Aquaculture Collaborative Innovation Center of Hubei Province, Wuhan Polytechnic University, Wuhan 430023, China; huxianqin@163.com

**Keywords:** cyclodextrin, prebaked bread, texture, mixolab, DSC

## Abstract

The effects of the addition of 0–3.0 wt% α-cyclodextrin (α-CD) and γ-cyclodextrin (γ-CD) on the quality of wheat flour as well as the texture and the aging of prebaked bread were evaluated. The addition of α-CD and γ-CD increased the ability of wheat flour to absorb water and shortened the times of dough formation and stabilization. Amylase activity slightly increased after using 2.0 and 3.0 wt% of α-CD and γ-CD, respectively. Moreover, the addition of α-CD and γ-CD increased the fermentation height and gas retention ability of dough. Dough samples containing 2.0 wt% α-CD and 3.0 wt% γ-CD showed the highest fermentation heights and gas retention volumes, respectively. Dough gas production increased with the addition of γ-CD. Gas production by dough samples containing more than 2.0 wt% α-CD exceeded that by samples in the control group. The results of the texture crumb of bread and specific volume tests revealed that the addition of 2.0 wt% α-CD and 3.0 wt% γ-CD reduced bread hardness and increased bread elasticity, resilience, and specific volume. The optimal α-CD and γ-CD contents were identified as 2.0 wt% and 3.0 wt%, respectively. The addition of 2.0 wt% α-CD and 3.0 wt% γ-CD delayed the aging of prebaked bread and reduced the hardness of prebaked bread during different weeks of storage, which may be due to decreasing the melting enthalpy of starch crystals. This work elucidated the mechanisms underlying the effects of CD addition on prebaked bread quality.

## 1. Introduction

Frozen dough production has become the preferred commercial bread production method with the development of refrigeration technology [1,2]. The use of frozen dough reduces the pressure on the industrial production of bread and saves processing time and storage costs. Freezing, however, has adverse effects on dough. For example, yeast death and ice crystal formation during freezing reduce the final bread specific volume and degrade bread texture [3].

Frozen prebaked bread technology was developed to meet the consumers’ need for fresh bread and avoid the adverse effects of dough freezing [4,5]. Frozen prebaked bread production mainly includes the processes of dough making, dough fermentation, dough baking (uncolored), frozen storage, and bread rebaking [6,7]. The requirements for complex processing techniques have resulted in problems with prebaked bread quality. For instance, prebaked bread has a lower specific volume and is more likely to harden during storage than conventional freshly baked bread. The processing parameters of different types of prebaked bread have been optimized, and the quality of prebaked bread has been improved [8,9]. However, the process parameters of prebaked bread production such as fermentation time, fermentation temperature, baking temperature, and baking time, etc. are often dependent on the characteristics and raw material composition of the final baked products. Thus, the quality problems of different types of prebaked bread products have yet to be resolved. 

In recent years, food additives including enzyme preparations, hydrocolloids, and polysaccharides have been used to improve prebaked bread quality and delay bread aging [10,11]. Nevertheless, most additives are poorly stable. For example, the ability of enzyme preparations to exert an antiaging effect on prebaked bread during storage after high-temperature denaturation remains problematic. Identifying a stable, healthy, and edible additive that can effectively improve the quality of dough and prebaked bread is important given the complexity of prebaked bread production. The rebaking stage, in particular, is highly complex. 

Several works have shown that the addition of 1.5% β-cyclodextrin (β-CD) to flour improved bread quality and delayed bread aging [12]. The complexation of the hydrophobic cavity of β-CD with amylose–lipid complexes inhibits bread aging by reducing starch retrogradation. In addition to β-CD, other common cyclodextrins are α-cyclodextrin (α-CD) and γ-cyclodextrin (γ-CD). However, the abilities of α-CD and γ-CD to improve prebaked bread quality and inhibit bread aging warrant further study. Cyclodextrins are cyclic molecules produced by amylase under the action of cyclodextrin glucosyltransferase [13]. The α/β/γ-CD contains 6, 7, 8 glucopyranose units, respectively, which are linked by a α-1,4 glycosidic bond to form a ring. Each CD molecule has a hydrophobic cavity and a hydrophilic outer ring and easily forms stable complexes with other substances, which are widely used in the food industry [14,15]. It is worth noting that α-CD and γ-CD are superior to β-CD in terms of lumen size, water solubility, bioavailability, and food safety. Therefore, α-CD and γ-CD have potentially greater application values in the food and pharmaceutical industries than β-CD. Additionally, the optimization of production processes is expected to extend the applications of α-CD and γ-CD in the future [16]. 

Therefore, in this study, the effects of the addition of 0–3.0 wt% α-CD and γ-CD (as a substitute) on flour powder parameters, fermentation performance, prebaked bread texture, and bread aging during the frozen storage were investigated. The results of this study will provide references for the application of α-CD and γ-CD in the bakery industry. 

## 2. Materials and Methods 

### 2.1. Materials

Flour was obtained by processing durum wheat (Xinmai26, Henan Province, China). Flour grade: first grade; Type of mill: MLU-202, Wuxi Buhler, China. The moisture, protein, and wet gluten contents of the flour were 11.5%, 11.88%, and 32.2%, respectively (calculated on a wet basis). Yeast was provided from Angel Yeast Co. Ltd. (Yichang, Hubei province, China) and α-CD and γ-CD were purchased from Shanghai Yuanye Bio-Technology Co. Ltd. (Shanghai, China)

### 2.2. Mixolab Test

The Mixolab test was performed in reference to previous studies with minor modifications [17,18]. Flour was mixed with different concentrations of α-CD and γ-CD and tested using a Chopin mixer (Mixolad, Chopin Technologies, France). A total of 75 g of flour was used in the Chopin test. The target consistency of the dough was 1.1 N/m. Specifically, the maximum torque of the dough reached 1.1 N/m (±0. 07 N/m), which is equivalent to a Brabender farinograph measurement of 500 BU. The procedure was divided into the following three stages: (1) Constant temperature stage: Temperature was maintained at 30 °C for 8 min; (2) Heating stage: Temperature was increased to 90 °C at the rate of 4 °C/min and maintained at 90 °C for 7 min; and (3) Cooling stage: Temperature was reduced to 50 °C at the rate of 4 °C/min and maintained at 50 °C for 5 min. The measurement stage required 45 min to complete.

### 2.3. Fermentation Test

The fermentation rheology test is used to evaluate the fermentation height and gas production capacity of the dough. Dough was prepared by using a Chopin Alveograph (RLTA10B, Chopin Technologies, Villeneuve-la-garenne, France) prior to testing through the following operation: 250 g of flour mixed with different concentrations of CDs was poured into the Chopin Alveograph. Then, 6 g of salt and 5 g of yeast powder were added to the flour. The dry ingredients were then mixed uniformly. Finally, water was added in accordance with the Mixolab test and Chopin test protocol. The dough was mixed for 8 min. After mixing, 315 g of the dough was transferred to a Chopin fermentation rheometer (F4, Chopin Technologies, Villeneuve-la-garenne, France) for testing [19]. The dough was tested at 28 °C for 3 h. The fermentation curve before 135 min was taken as the final analytical result. 

### 2.4. Prebaked Bread Production

The prebaked bread production method used in this study was based on a previously reported method and was slightly modified in accordance with the actual situation [20]. The bread formula was as follows: 1000 g of flour, 0.5% w/w dry yeast, 1.6% w/w salt, 8% w/w butter, 6% w/w sugar, and 0–3.0 w/w% α/γ-CD. An automatic mixer (SPI 11, PETRIN SPIRALE LAB0, Villeneuve-la-garenne, France) was used to prepare the dough. Flour, yeast, sugar, and CD were mixed first. Then, the dry ingredients were mixed with water. The initial water temperature was maintained at 8 °C to 9 °C with ice. Butter was added in accordance with the state of dough formation. Salt was added to the dough after the completion of the gluten network formation (a good gluten film appears). The dough was divided into buns with weights of 80 g. Then, a pressure stick was used to vent the dough as well as squeezing it back and forth twice by allowing the dough to stand at room temperature for 20 min. The dough was then fermented at 30 °C and 85% RH for 60 min. Gas was then discharged from the dough. Next, the dough was fermented for 60 min. After fermentation, the dough was baked in an oven (Backcombi, Germany) at an upper temperature of 210 °C and a bottom temperature of 180 °C for 10 min under steam conditions. After baking, bread samples were removed from the oven, cooled at room temperature for 1 h, and then placed in a freezer at −35 °C for 40 min. The bread was then transferred to a freezer at −18 °C for three days. Next, the bread was thawed for 20 min at 30 °C. Finally, it was baked at a top temperature of 210 °C and a bottom temperature of 180 ° C for 10 min.

### 2.5. Effects of the Addition of 0–3.0 wt% α-CD and γ-CD on the Textural Properties of Prebaked Bread Crumb 

This experimental method had minor adjustments with reference to the previous test methods [12]. The texture of rebaked bread crumb was characterized (frozen for three days). A bread crumb with dimensions of 20 mm × 20 mm was collected and tested immediately (TA. XT plus, Stable Micro Systems, Shanghai, China) with the premeasuring speed of 1 mm/s, test speed of 3 mm/s, posttest speed of 3 mm/s, and compression displacement of 50%. The experiments were repeated six times.

### 2.6. Specific Volume Test and Slice Structure Test

Bread specific volume was quantified using a food volume meter (BVW-L370, Perten, Sweden). Six samples were tested per group. The average of the results was calculated. The bread slice structure test was slightly adjusted based on previous test methods [21]. A bread slice of about 12 mm width was cut from the center of the bread and subjected to image analysis (Calibre Control International Ltd., Warrington, England).

### 2.7. Effects of the Addition of 2.0 wt% α-CD and 3.0 wt% γ-CD on the Textural Properties of Prebaked Bread Crumb After 1, 2, or 3 Weeks of Frozen Storage

The optimal α-CD and γ-CD contents were identified as 2.0 and 3.0 wt%, respectively. Thus, flour was modified with 2.0 wt% α-CD or 3.0 wt% γ-CD. Prebaked bread was prepared in accordance with the method described in Section 2.4. Bread was rebaked after storage at −18 °C for 1, 2, or 3 weeks. The texture of the prebaked bread was tested by using the method described in Section 2.5. 

### 2.8. Differential Scanning Calorimetry Test

The differential scanning calorimetry test (DSC, Q2000, TA, USA) was performed in reference to previous studies with minor adjustments [12,22]. Bread frozen for 1, 2, or 3 weeks was removed from storage and rebaked. Bread crumb with dimensions of 20 mm × 20 mm were collected from the rebaked bread, freeze-dried for 48 h, and then pulverized to pass through a 160 mesh sieve. The test conditions were as follows: heating rate of 10.0 °C/min, nitrogen flow rate of 20 mL/min, and scanning temperature range of 25–230 °C. The thermal effect curve of the sample was acquired. The initial transition temperature (T_0_), peak phase temperature (Tp), and phase transition enthalpy (ΔH) were recorded.

### 2.9. Statistical Analysis

Experiments were performed with a completely randomized design. All experiments were repeated, and the means and standard deviations were calculated. Origin (OriginPro 9.0 64-bit, OriginLab, Hampton City, MA, USA) was used to plot the raw data. Analysis of variance was performed, and the results were separated using Duncan’s multiple range test (*p* < 0.05) by using Statistical Package for the Social Sciences software.

## 3. Results and Discussion

### 3.1. Mixolab Test

In the mixer, the water absorption index reflects the characteristics of the water absorption of the flour. As shown in Table 1, the water absorption rates of the groups containing α-CD and γ-CD were higher than those of the control group and showed similar trends. Th water absorption rate increased from 61.3 wt% to nearly 65.0 wt% with the addition of 3.0 wt% α-CD or γ-CD. During the dough formation process, the protein absorbs a large amount of water to form a gluten network. Studies have shown that water absorption by protein in flour may be affected by the interaction between CDs and proteins through hydrogen bonding [23]. In this test, as the amount of cyclodextrin added increased, the water absorption of the dough increased, which may be due to the hydrogen bonding between the cyclodextrins and proteins. In addition, α-CD and γ-CD are hydrophilic in the external sections while the internal hydrophobic cavities of α-CD and γ-CD can form stable inclusion complexes with water molecules, thus explaining the increased water absorption of the dough [15]. Ft represents the formation time of the dough. The dough formation time was drastically shortened when the α-CD and γ-CD concentrations were increased. 

Specifically, the dough formation time decreased from 10.2 min to nearly 8.4 min when the α-CD and γ-CD content reached 3.0 wt%. The addition of α-CD and γ-CD increased the dough moisture content, thus promoting water absorption by protein in the flour, and accelerating the gluten network formation. These effects reduced dough formation time. 

The mixing index reflects the stability of the dough. Similarly, dough stabilization time decreased with the addition of α-CD or γ-CD. Dough stabilization time decreased from 10.5 min to 9.1 and 8.7 min with the addition of 3.0 wt% α-CD or 3.0 wt% γ-CD, respectively. Dough stability reflects the resistance of dough to mechanical whipping, and the reduction in dough toughness with increasing moisture content shortens the dough stabilization time [24]. Sufficient formation and settling times are prerequisites of dough quality. The Mixolab test results obtained in the present study indicate that α-CD and γ-CD content must be controlled in practical applications. Amylase content and activity play an important role in dough fermentation, bread volume, and texture [25]. The amylase index reflects the properties of amylase-degrading starch. In Mixolab tests, high amylase indexes are indicative of low amylase activity. The amylase index decreased from 9 to 8 when the α-CD content exceeded 2.0 wt% or the γ-CD content exceeded 3.0 wt%. This result indicates that the amylase activity slightly intensified. This effect may improve the quality of prebaked bread as shown in the subsequent baking test. 

### 3.2. Fermentation Test

**Figure 1:**
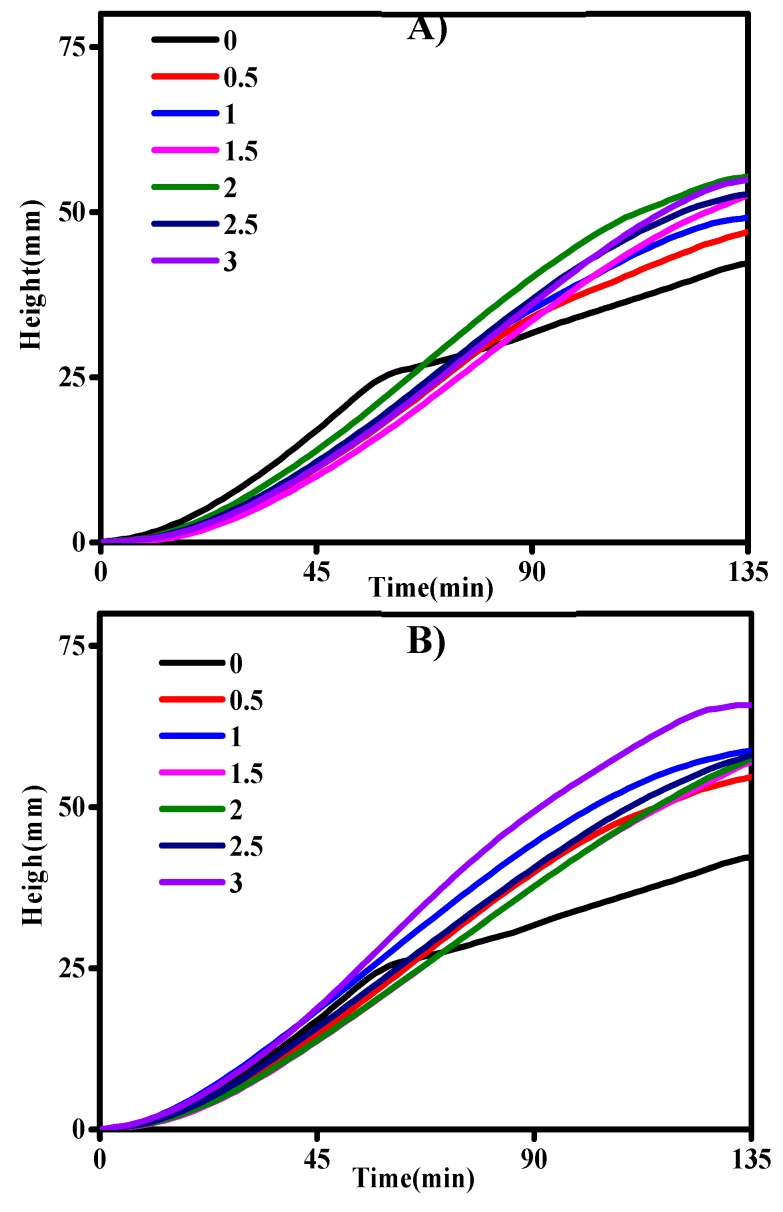
Effect of the addition of 0–3.0 wt% α-CD (**A**) and 0–3.0 wt% γ-CD (**B**) on dough fermentation height as observed with the F4 fermentation rheometer. Dough was fermented at 28 °C for 135 min.

As shown in Figure 1A,B, the dough fermentation height of the groups modified with α-CD and γ-CD exceeded that of the control group at the fermentation time of 135 min. The highest dough fermentation heights were obtained with the addition of 2.0 wt% α-CD and 3.0 wt% γ-CD. Dough fermentation height is highly influenced by a combination of factors including dough strength, gas production, and gluten network structure [26]. In the present work, however, Mixolab test results showed that dough stability decreased. This reduction indicates that the increase in dough height may be related to gas production (Table 2). 

Gas production by the group containing 0.5 wt% to 1.5 wt% α-CD was slightly reduced relative to that by the control group, whereas that by the group modified with 2.0 wt% to 3.0 wt% α-CD increased. In contrast to the addition of α-CD, 0.5 wt%–3.0 wt% γ-CD increased dough gas production. Dough gas production is primarily related to yeast behavior during dough fermentation. The Mixolab tests showed that amylase activity increased with the addition of 2.0 wt% α-CD and 3.0 wt% γ-CD. This result implies that the yeast-available sugar content of the dough increased. In general, an elevation in dough sugar content promotes yeast fermentation and gas production. The addition of 0.5 wt%–3.0 wt% α-CD and γ-CD increased the retained gas volume and gas retention ratio of the dough samples. Overall, the group modified with 2.0 wt% α-CD and 3.0 wt% γ-CD showed the largest gas retention volume. 

**Figure 2:**
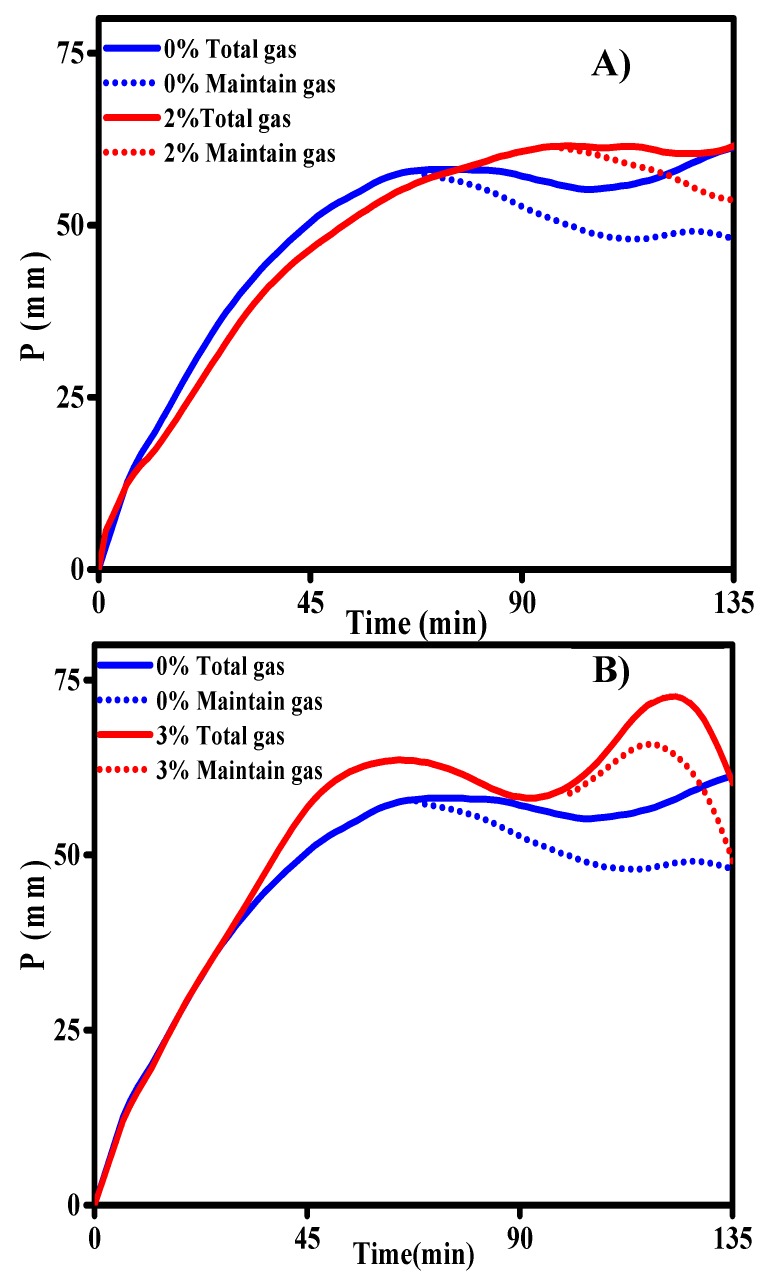
Dough fermentation curves of the 2.0 wt% α-CD and control groups (**A**) and 3.0 wt% γ-CD and control groups (**B**). Fermentation curves were obtained using the F4 fermentation rheometer. The solid line represents the total gas production and the dashed line represents the retained gas volume.

The fermentation gas curves of the 2.0 wt% α-CD, 3.0 wt% γ-CD, and control groups are shown in Figure 2A,B. The dough must retain the gas produced during fermentation and baking for a sufficiently long period to produce a well-expanded loaf of bread with an even crumb texture [27]. The addition of CDs, particularly the addition of 2.0 wt% α-CD and 3.0 wt% γ-CD, enhanced the gas retention capacity of the dough. This effect would have positive implications for the quality of prebaked bread products.

### 3.3. Effects of the Addition of 0–3.0 wt% α-CD and γ-CD on the Textural Properties of Prebaked Bread 

Texture analyzers are one of the most commonly used methods for evaluating bread quality. Important indicators of the texture test results mainly include bread hardness, elasticity, and resilience. Resilience refers to the ability of a sample to rebound during the first compression process. That is, the ratio of the elastic energy released by the returned sample to the energy consumption of the probe during compression during the first compression cycle. 

**Figure 3:**
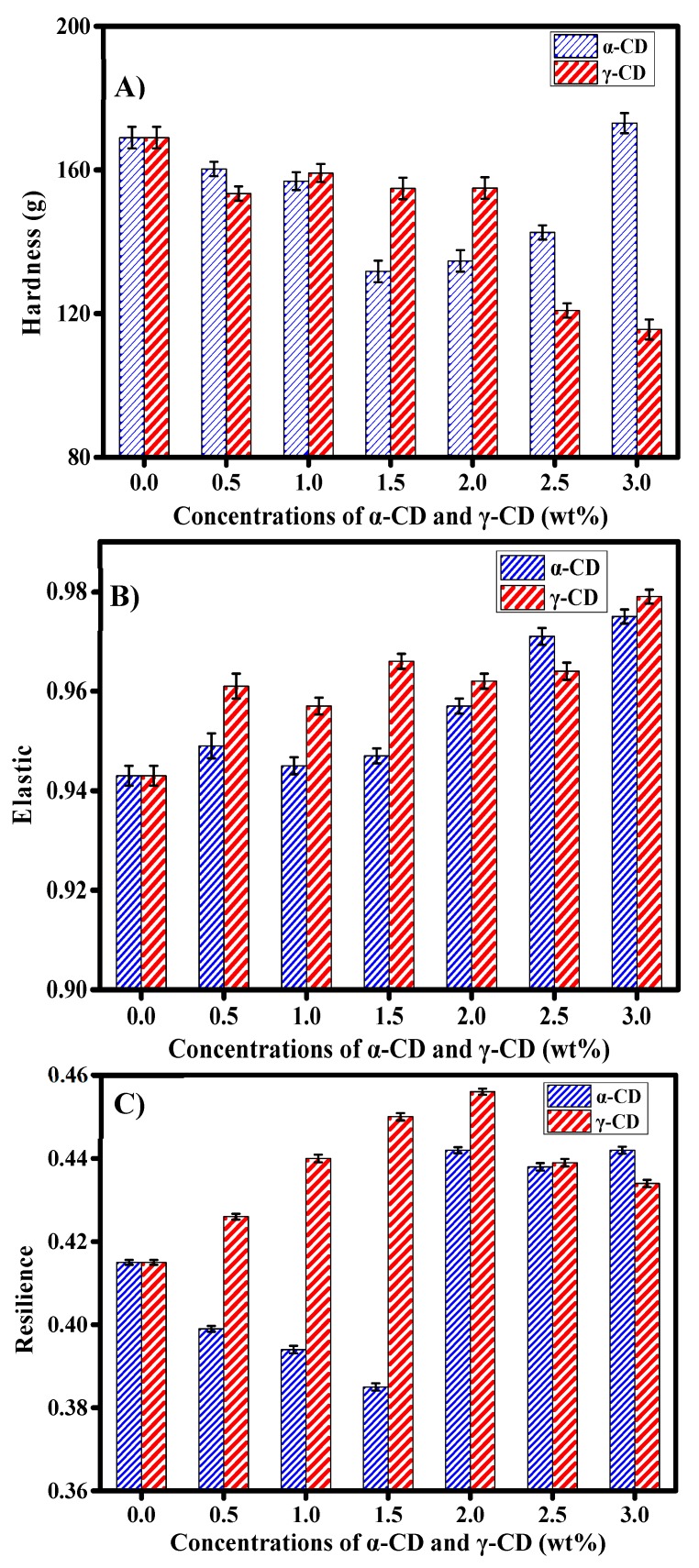
Effects of the addition of α-CD and γ-CD on the hardness (**A**), elasticity (**B**), and resilience (**C**) of frozen prebaked bread (frozen for three days). Bread samples were cut into 20 mm × 20 mm × 20 mm pieces and placed under the P/100 probe. Samples were tested three times. The average of three measurements was calculated. Each group was tested six times in parallel.

As shown in Figure 3A, the addition of 0.5–2.5 wt% α-CD or 0.5–3.0 wt% γ-CD reduced the hardness of the prebaked bread. However, the addition of 3.0 wt% α-CD increased the bread hardness. In comparison, the addition of 2.0 wt% α-CD or 3.0 wt% γ-CD group had the lowest hardness value. Figure 3B showed that the addition of γ-CD increased the bread elasticity. Like α-CD, the elasticity of the bread increased significantly when the amount added reached 2.0 wt%. From Figure 3C, it can be depicted that the addition of 0.5–1.5 wt% of the α-CD reduced the resilience of the prebaked bread; when the amount of α-CD added reached 2.0 wt%, the resilience of the prebaked bread increased. Moreover, the addition of 0.5–3.0 wt% γ-CD increased the resilience of the prebaked bread (Figure 3C). 

Overall, the addition of 2.0 wt% α-CD or 3.0 wt% γ-CD significantly improved the texture of the prebaked bread. The quality of prebaked bread is mainly determined in two stages: dough making and bread storage. In this sense, previous research has pointed out that the addition of compounds capable of interacting with water in wheat flour can affect the quality of the final baked product [28,29]. 

The results of the Mixolab test showed that the addition of α-CD and γ-CD affected the textural properties of rebaked bread by increasing the water absorption capacity of wheat flour. Likewise, the fermentation test results revealed that the addition of 2.0 wt% α-CD and 3.0 wt% γ-CD could increase the gas production and gas retention capacity of the dough samples; these effects may partly account for the improvement in the hardness and elasticity of rebaked bread [30]. 

During storage, moisture migrates from the bread core to the bread crust and finally to the external environment of the bread. Moisture loss increases bread hardness and reduces bread elasticity [31]. CDs have good water-holding capacity, and different CDs have different water-binding abilities [32,33]. This property may inhibit the migration of moisture during prebaked bread storage and the deterioration of bread quality. On the other hand, the deterioration of prebaked bread quality during storage is also related to bread aging, which will be discussed in the following sections [34]. 

### 3.4. Specific Volume and Slice Structure Test

**Figure 4:**
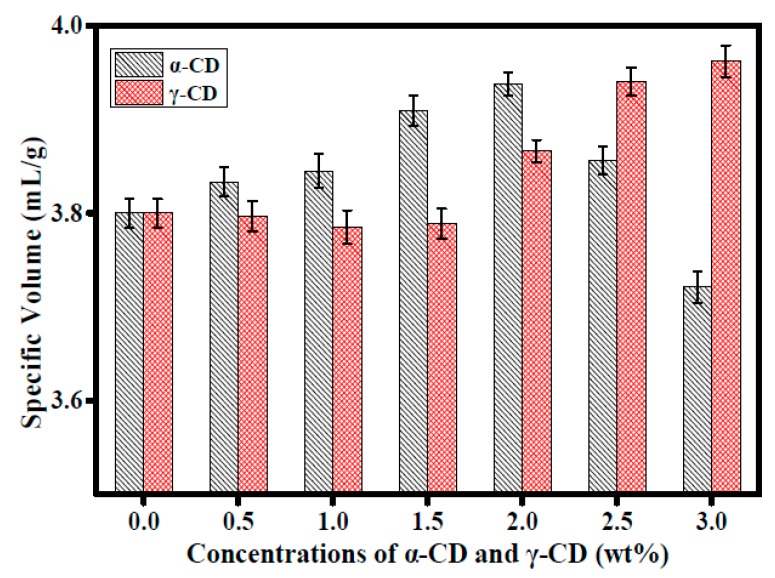
Effects of the addition of 0–3.0 wt% α-CD and γ-CD on the specific volume of frozen prebaked bread (frozen for three days). Samples were tested three times. The average of three measurements was calculated. Each group was tested six times in parallel.

**Figure 5:**
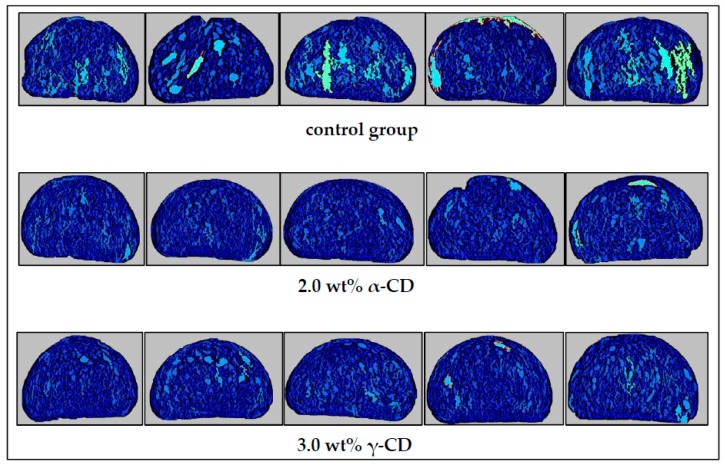
Slice structure of the prebaked bread samples in the control group, 2.0 wt% α-CD group, and 3.0 wt% γ-CD group (frozen for three days). Each group was tested six times in parallel with three repetitions. Three images were taken for comparison.

As it showed in Figure 4, the addition of 0.5 wt%–2.0 wt% α-CD increased the specific volume of prebaked bread. The specific volume of the 3.0 wt% α-CD group was lower than that of the control group. The specific volume of prebaked bread increased with the addition of γ-CD and reached the highest value with the addition of 3.0 wt% γ-CD. The tissue structures of the 2.0 wt% α-CD, 3.0 wt% γ-CD, and control groups are shown in Figure 5. The specific volume of prebaked bread is related to the numbers and sizes of its internal pores. In general, large numbers and volumes of pores are indicative of high specific volumes, so pore production is closely related to dough fermentation [35]. 

The addition of 2.0 wt% α-CD and 3.0 wt% γ-CD increased the fermentation height and gas production of prebaked bread. These results revealed that the addition of CDs changed the amounts and sizes of the pores in the dough. These effects may account for the increment in the specific volume of the prebaked bread samples. For instance, the photographs of the prebaked bread slices (Figure 5) revealed that the addition of 2.0 wt% α-CD and 3.0 wt% γ-CD altered the distribution of pores and reduced the number of large pores. The small number of large pores and the uniformity of pore size in the tissue structures of the prebaked bread slices indicated that bread tissue was fine and smooth [36], thus showing that the addition of CDs improved the fineness of the tissue of the prebaked bread.

### 3.5. Effects of the Addition of 2.0 wt% α-CD and 3.0 wt% γ-CD on the Textural Properties of Prebaked Bread Subjected to 1, 2, or 3 Weeks of Frozen Storage

The optimal α-CD and γ-CD contents were identified as 2.0 and 3.0 wt%, respectively, on the basis of the effects of different concentrations of α-CD and γ-CD (0–3.0 wt%) on the textural properties of prebaked bread. The effect of the two CDs on the quality of prebaked bread subjected to different durations of frozen storage time was further studied. As shown in Table 3, the prebaked bread quality decreased with the elapse of frozen storage. Specifically, bread hardness increased, whereas bread elasticity and recovery decreased. The addition of 2.0 wt% α-CD and 3.0 wt% γ-CD improved the prebaked bread texture. The addition of CD reduced hardness and increased the elasticity and resilience of bread samples stored for the same duration. Frozen storage adversely affected prebaked bread quality. The addition of 2.0 wt% α-CD and 3.0 wt% γ-CD could effectively alleviate this negative effect. Along this line, a previous study showed that the quality of prebaked bread changed mainly during the early stages of frozen storage (1 week) and that the texture of prebaked bread negligibly changed with prolonged frozen storage [8]. The inconsistency between the results of the present study and the previous one may be attributed to differences between the bread composition and baking processes and indicates that different types of prebaked bread undergo different changes during storage.

Moreover, the addition of 2.0 wt% α-CD and 3.0 wt% γ-CD delayed the hardening of prebaked bread and decelerated the rate of the decline in the elasticity and resilience of prebaked bread. In particular, the addition of 3.0 wt% γ-CD significantly improved the quality of prebaked bread. Overall, the addition of the two CDs slowed down the degradation of the prebaked bread texture. For instance, studies have confirmed that the addition of β-CD can delay the aging behavior of bread [12]. In this study, the improvement in prebaked bread texture with the addition of 2.0 wt% α-CD and 3.0 wt% γ-CD may be related to the delayed aging of prebaked bread.

### 3.6. Differential Scanning Calorimetry (DSC) Test Analysis 

The DSC results obtained in this study (Table 4) showed that the initial temperature, peak temperature, and enthalpy of the prebaked bread sample increased with the lapse of freezing time. During the cooling process, the starch in the prebaked bread is regenerated, which usually leads to an increase in the hardness of the bread and a decrease in elasticity. Starch retrogradation involves short-term and long-term retrogradation. Short-term retrogradation is mainly related to the behavior of amylose gel formation, which usually occurs within a few hours after the end of baking. Long-term retrogradation is related to amylopectin recrystallization, which usually takes several weeks [37]. Amylose and amylopectin recrystallize in prebaked bread during storage (3 weeks, −18 °C), rebaking, and cooling (1 d). 

In this study, the increase in the initial temperature, peak temperature, and enthalpy of prebaked bread samples with prolonged frozen storage time indicates that starch retrogradation occurred during freezing.

The transition temperature of samples containing 2.0 wt% α-CD and 3.0 wt% γ-CD increased. Moreover, as can be seen in Table 4, the initial transition temperature of the prebaked samples was 50 °C–60 °C and the peak phase transition temperature of the samples fell in the range of 100 °C–110 °C. As is well known, amylopectin has a slow regenerative rate and weak crystal structure. Its melting temperature is close to the temperature of starch gelatinization, but the melting temperature of the amylose crystal is higher than 100 °C [38,39]. 

The addition of 2.0 wt% α-CD and 3.0 wt% γ-CD increased the initial and peak phase transition temperatures of prebaked bread most likely because the hydrophobic cavity of the CDs form a complex with amylose–lipid complexes. This phenomenon increased the melting temperature of CDs. The inconsistent effect of α-CD and γ-CD on phase transition temperature may be attributed to variations in the amounts of CDs and the sizes of the hydrophobic cavities of the CDs. 

**Figure 6:**
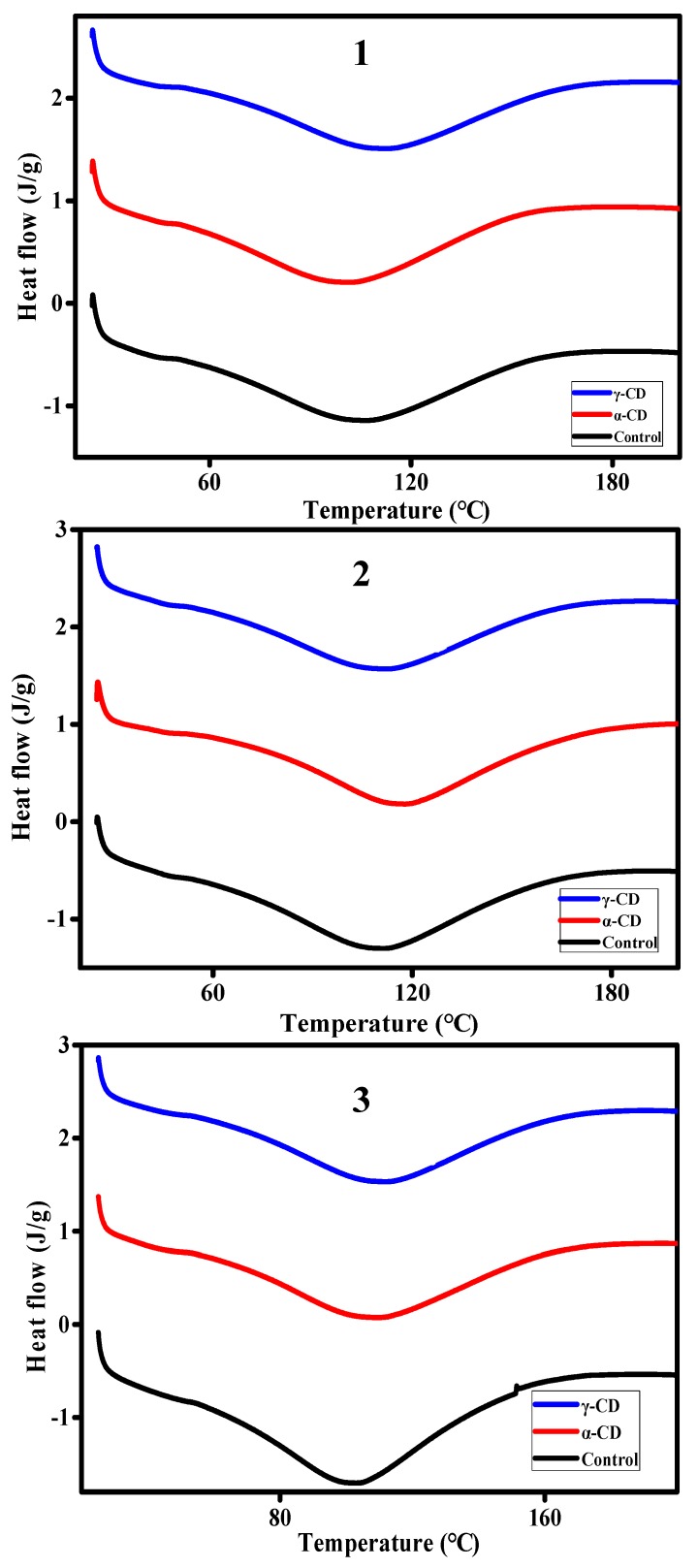
Typical DSC test curve of prebaked bread samples in the control, 2.0 wt% α-CD, and 3.0 wt% γ-CD groups that had been frozen for 1(1), 2(2), or 3(3) weeks.

Comparing the average ΔH values of bread samples frozen for three weeks revealed that the average ΔH values of the 2.0 wt% α-CD and 3.0 wt% γ-CD groups were 215.6 and 199.7 J/g, respectively, whereas that of the control group was 238.8 J/g. This result indicates that the addition of 2.0 wt% α-CD and 3.0 wt% γ-CD affected starch retrogradation in prebaked bread samples. Figure 6 shows the typical DSC curves of prebaked bread in different freezing cycles. As visually depicted from Figure 6, the absorption peak of the blank group was larger than the absorption peaks of the 2.0% α-CD and 3.0% γ-CD group, and was particularly obvious when the samples were frozen for three weeks. The addition of β-CD inhibited starch recrystallization in bread through the formation of amylose–lipid–β-CD complexes [12]. The formation of amylose–lipid–cyclodextrin complexes was partially responsible for reducing enthalpy by hindering the rearrangement of amylose into crystals [12]. The reduction in the enthalpy of prebaked bread samples with the addition of 2.0 wt% α-CD and 3.0 wt% γ-CD indicates that the number of starch crystals decreased, thus observing an inhibition in starch retrogradation after the addition of α-CD and γ-CD. As previously observed, starch recrystallization is often accompanied by water migration [40]. However, the good water-holding capacity of α-CD and γ-CD may inhibit moisture loss during storage and is indicative of a reduction in the degree of starch retrogradation. The results of the DSC test also suggested that the improvement in the textures of prebaked breads containing 2.0 wt% α-CD and 3.0 wt% γ-CD and subjected to different durations of frozen storage might be associated with a decrease in enthalpy. 

## 4. Conclusions

The results of this study showed that the addition of α-CD and γ-CD to flour increased the water absorption capacity of the dough. This effect shortened the dough formation time and reduced dough resistance to mechanical whipping. Insufficient dough formation and settling times are detrimental to dough quality. The appropriate α-CD and γ-CD contents should be considered in the actual industrial production of frozen prebaked bread. Moreover, the addition of suitable amounts of α-CD and γ-CD improved dough fermentation performance as confirmed by the texture, specific volume, and slice structure properties of the final prebaked bread samples containing α-CD and γ-CD. The results of the DSC test showed that the addition of 2.0 wt% α-CD and 3.0 wt% γ-CD drastically reduced the enthalpy of prebaked bread samples. Thus, similar to β-CD, α-CD and γ-CD may exert antiaging effects by inhibiting starch retrogradation. Finally, the addition of 2.0 wt% α-CD and 3.0 wt% γ-CD drastically improved the quality of dough and prebaked bread and could extend the shelf life of prebaked bread.

## Figures and Tables

**Table 1:** Effects of different α-CD and γ-CD contents (0–3.0 wt%) on wheat flour quality.

CD	(*wt* %)	0	0.5	1.0	1.5	2.0	2.5	3.0
**α**	Wa (%)	61.3 ± 0.1 ^a^	62.2 ± 0.2 ^b^	62.6 ± 0.1 ^c^	63.5 ± 0.2 ^d^	64.0 ± 0.1 ^e^	64.5 ± 0.1 ^f^	65.0 ± 0.1 ^g^
	Ft (min)	10.2 ± 0.1 ^a^	9.8 ± 0.1 ^b^	9.6 ± 0.1 ^b^	9.0 ± 0.1 ^c^	9.2 ± 0.0 ^c^	8.5 ± 0.1 ^d^	8.4 ± 0.1 ^d^
	St (min)	10.5 ± 0.1 ^a^	10.3 ± 0.1 ^b^	10.2 ± 0.0 ^b^	10.0 ± 0.1 ^c^	9.7 ± 0.1 ^d^	9.7 ± 0.1 ^d^	9.1 ± 0.1 ^e^
	AI	9 ± 0	9 ± 0	9 ± 0	9 ± 0	8 ± 0	8 ± 0	8 ± 0
**γ**	Wa (%)	61.3 ± 0.1 ^a^	62.3 ± 0.1 ^b^	62.8 ± 0.1 ^c^	63.2 ± 0.2 ^d^	63.5 ± 0.1 ^e^	64.5 ± 0.2 ^f^	65.2 ± 0.1 ^g^
	Ft (min)	10.2 ± 0.1 ^a^	10.0 ± 0.1 ^b^	9.5 ± 0.1 ^c^	9.0 ± 0.1 ^d^	8.8 ± 0.0 ^e^	8.5 ± 0.1 ^f^	8.3 ± 0.1 ^g^
	St (min)	10.5 ± 0.1 ^a^	10.5 ± 0.1 ^a^	10.1 ± 0.1 ^b^	10.0 ± 0.1 ^b^	9.4 ± 0.1 ^c^	9.2 ± 0.1 ^c^	8.7 ± 0.2 ^d^
	AI	9 ± 0	9 ± 0	9 ± 0	9 ± 0	9 ± 0	9 ± 0	8 ± 0

Note: Different letters in the same row indicate significant differences (*p* < 0.05), and no or the same letters indicate no significant differences. Wa = Water absorption (%), Ft = Formation time (min), St = Stabilization time (min), AI = Amylase index.

**Table 2:** Effects of different concentrations of α-CD and γ-CD (0–3.0 wt%) on the dough volume, total gas, and maintain gas volume (135 min).

CD	wt%	0	0.5	1.0	1.5	2.0	2.5	3.0
α	Maintain gas (mL)	1038 ± 4 ^a^	1100 ± 3 ^b^	1118 ± 4 ^c^	1151 ± 5 ^d^	1226 ± 4 ^e^	1167 ± 5 ^f^	1190 ± 4 ^g^
	Total gas (mL)	1227 ± 3 ^a^	1200 ± 5 ^b^	1219 ± 3 ^c^	1225 ± 4 ^c^	1363 ± 2 ^d^	1276 ± 6 ^e^	1288 ± 3 ^f^
	Ratio (%)	84.6 ± 0.4 ^a^	91.7 ± 0.3 ^b^	91.7 ± 0.3 ^b^	93.9 ± 0.4 ^c^	90.0 ± 0.3 ^d^	91.4 ± 0.4 ^b^	92.4 ± 0.3 ^e^
γ	Maintain gas (mL)	1038 ± 4 ^a^	1199 ± 5 ^b^	1206 ± 4 ^b^	1220 ± 3 ^c^	1235 ± 6 ^d^	1238 ± 3 ^d^	1294 ± 4 ^e^
	Total gas (mL)	1227 ± 5 ^a^	1319 ± 5 ^b^	1340 ± 6 ^c^	1360 ± 3 ^d^	1365 ± 4 ^d^	1375 ± 4 ^e^	1396 ± 3 ^f^
	Ratio (%)	84.6 ± 0.3 ^a^	90.9 ± 0.4 ^b^	90.0 ± 0.3 ^c^	89.8 ± 0.2 ^c^	90.3 ± 0.4 ^c^	90.0 ± 0.2 ^c^	92.7 ± 0.3 ^d^

Different letters in the same row indicate significant differences (*p* < 0.05), and no or the same letters indicate no significant differences.

**Table 3:** Effects of the addition of 2.0 wt% α-CD and 3.0 wt% γ-CD on the texture of prebaked bread samples that had been frozen for 1, 2, or 3 weeks.

Time (W)	-CD	Hardness (g)	Elastic	Resilience
1	Control group	180.2 ± 2.3 ^a^	0.942 ± 0.007 ^a^	0.403 ± 0.003 ^a^
	2.0 *wt*% α-CD	150.4 ± 2.5 ^b^	0.963 ± 0.004 ^b^	0.424 ± 0.003 ^b^
	3.0 *wt*% γ-CD	114.3 ± 3.2 ^c^	0.980 ± 0.008 ^c^	0.433 ± 0.002 ^c^
2	Control group	215.3 ± 3.1 ^d^	0.912 ± 0.005 ^d^	0.382 ± 0.005 ^d^
	2.0 *wt*% α-CD	173.4 ± 2.3 ^ae^	0.946 ± 0.006 ^a^	0.417 ± 0.002 ^e^
	3.0 *wt*% γ-CD	135.2 ± 3.5 ^f^	0.951 ± 0.003 ^a^	0.426 ± 0.004 ^b^
3	Control group	248.5 ± 1.8 ^g^	0.874 ± 0.005 ^e^	0.355 ± 0.002 ^f^
	2.0 *wt*% α-CD	208.3 ± 2.1 ^d^	0.921 ± 0.005 ^d^	0.393 ± 0.004 ^g^
	3.0 *wt*% γ-CD	168.1 ± 1.8 ^e^	0.942 ± 0.003 ^a^	0.403 ± 0.003 ^a^

Different letters in the same column indicate significant differences (*p* < 0.05), and no or the same letters indicate no significant differences.

**Table 4:** Effects of the addition of 2.0 wt% α-CD and 3.0 wt% γ-CD on the enthalpy of prebaked bread samples that had been frozen for 1, 2, or 3 weeks.

Time (W)	-CD	T_0_ (°C)	T_p_ (°C)	Δ_H_ (J/g)
1	Control group	49.1 ± 1.2 ^a^	104.6 ± 1.2 ^a^	190.5 ± 2.6 ^a^
	2.0 *wt*% α-CD	52.2 ± 1.1 ^b^	105.2 ± 0.8 ^ab^	186.4 ± 1.7 ^ab^
	3.0 *wt*% γ-CD	53.2 ± 0.9 ^bc^	105.9 ± 0.7 ^abc^	179.8 ± 2.9 ^b^
2	Control group	53.0 ± 1.4 ^bc^	105.4 ± 0.7 ^ab^	205.1 ± 3.1 ^c^
	2.0 *wt*% α-CD	54.1 ± 1.3 ^bcd^	106.7 ± 0.7 ^bc^	190.8 ± 2.4 ^a^
	3.0 *wt*% γ-CD	55.5 ± 1.1 ^bcd^	107.1 ± 0.8 ^cd^	184.6 ± 1.8 ^ab^
3	Control group	55.5 ± 0.9 ^bcd^	106.7 ± 0.6 ^bc^	238.8 ± 3.6 ^d^
	2.0 *wt*% α-CD	56.1 ± 0.8 ^cd^	108.2 ± 0.5 ^d^	215.6 ± 3.7 ^e^
	3.0 *wt*% γ-CD	57.7 ± 1.2 ^d^	108.4 ± 1.1 ^d^	199.7 ± 3.2 ^c^

Different letters in the same column indicate significant differences (*p* < 0.05), and no or the same letters indicate no significant differences.
